# Focused Ultrasound-Triggered Burst Release and Enrichment of Engineered Bacteria for Tumor Therapy

**DOI:** 10.34133/bmr.0354

**Published:** 2026-04-16

**Authors:** Jinhee Yoo, Yunhee Hwang, Mihyeon Park, Honghyeon Ha, Myeong Ryeol Choi, Sung In Lim, Byullee Park, Yong Joo Ahn, Won Jong Kim, Gyoo Yeol Jung, Hyung Ham Kim

**Affiliations:** ^1^ Department of Electrical Engineering, Pohang University of Science and Technology, Pohang 37673, Republic of Korea.; ^2^ Department of Chemical Engineering, Pohang University of Science and Technology, Pohang 37673, Republic of Korea.; ^3^ Department of Chemistry, Pohang University of Science and Technology, Pohang 37673, Republic of Korea.; ^4^ Department of IT Convergence, Pohang University of Science and Technology, Pohang 37673, Republic of Korea.; ^5^ Medical Science and Engineering, Pohang University of Science and Technology, Pohang 37673, Republic of Korea.; ^6^ Department of Chemical Engineering, Pukyong National University, Busan 48513, Republic of Korea.; ^7^ Department of Biophysics, Institute of Quantum Biophysics, Sungkyunkwan University, Suwon 16419, Republic of Korea.

## Abstract

Ultrasound has been widely used in bacteria-based tumor therapy for targeted drug delivery due to its noninvasive ability to control bacteria. However, current approaches achieve only gradual release of therapeutic agents and rely on limited natural homing ability, resulting in poor treatment efficacy. Here, we demonstrate that focused ultrasound (FUS) can simultaneously induce burst release within tumors and local enrichment of therapeutics, enhancing precise and controllable spatiotemporal drug delivery efficiency within tumor microenvironments in living-therapeutic biomaterial system. We achieved FUS-triggered bacterial burst release by coexpressing gas vesicles (GVs), protein nanostructures that induce cavitation, and tumor necrosis factor-related apoptosis-inducing ligand (TRAIL), which increases antitumor efficacy. Furthermore, we show that FUS enhances enrichment of therapeutics at the tumor site by manipulating bacteria toward tumor vessel walls and increasing tumor permeability via sonopermeation. In a mouse model, we demonstrated clinical potential by achieving a 40% reduction in tumor volume using probiotic *Escherichia coli* Nissle (EcN) 1917 with a short 2-min FUS exposure and no exogenous agents. This work shows that physical ultrasound control provides an intuitive, highly efficient, and easily applicable biohybrid approach to bacteria-based tumor therapy, offering a simple and widely accessible strategy that can be broadly adapted to diverse living biomaterial systems.

## Introduction

Despite advances in tumor treatment, there remains a critical need for therapies that can selectively target tumor tissues. Bacteria-based tumor therapy addresses this challenge by exploiting the unique ability of bacteria to selectively colonize tumor sites, particularly within hypoxic environments [1]. This natural targeting ability minimizes damage to healthy tissues. Bacteria also induce an inflammatory response within the tumor microenvironment by activating immune cells, such as macrophages and dendritic cells [2]. Furthermore, bacteria are amenable to synthetic biology, enabling the addition of diverse functional capabilities [1,3]. Despite these advantages, bacterial tumor therapy still faces limitations in therapeutic efficacy and carries risks such as sepsis [4]. To overcome these challenges, recent studies have explored the control of bacterial behavior using external triggers such as focused ultrasound (FUS) [3,5–8].

FUS provides a noninvasive and spatiotemporally precise modality to control bacterial behavior in deep tissues, which has led to its widespread use in bacteria-based biohybrid tumor therapy. For instance, ultrasound-induced heating can activate heat-sensitive promoters to trigger localized therapeutic protein release [9–11]. Bacteria engineered to express gas vesicles (GVs), protein nanostructures, enable ultrasound-mediated cavitation to enhance tumor disruption [12,13]. Catalase produced by bacteria can work synergistically with ultrasound-responsive sensitizers to generate reactive oxygen species (ROS) under ultrasound activation, leading to cancer cell death [14,15]. Nevertheless, these approaches have primarily relied on therapeutic agents gradually secreted by bacteria and depend on bacterial inherent homing ability for tumor targeting, resulting in limited treatment efficacy. In addition, previous ultrasound strategies pose challenges for clinical studies because they require long exposure times and multiple treatments [9,14], utilize laboratory strains that may not be optimal for biomedical applications [9,13–15], or depend on exogenous agents [12,13].

To more concretely define these practical limitations, many representative ultrasound-controlled bacterial therapy protocols reported to date typically involve ultrasound exposure durations on the order of 10 to 30 min per treatment session and/or require repeated insonation across multiple sessions (often ≥2 treatments) [9,14–16]. In many cases, these protocols further rely on auxiliary components, such as nanosonosensitizers or complex multi-element transducer systems, to achieve sufficient biological activity [12–16]. In contrast, achieving therapeutic efficacy using a single ultrasound session with an exposure duration within 2 min, without the use of exogenous agents, has remained a key unmet practical challenge.

**Fig. 1.**
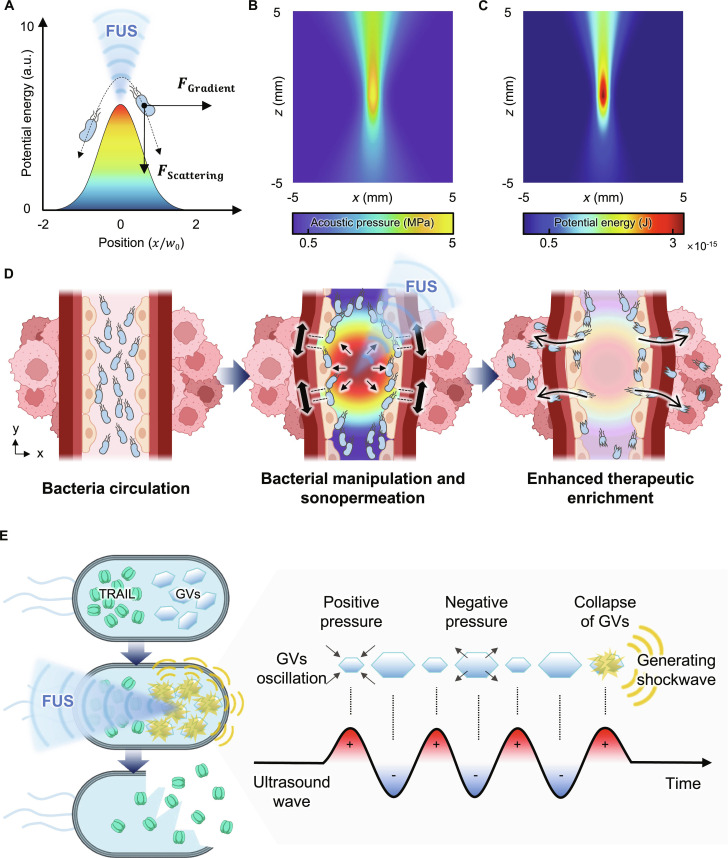
Schematic of FUS-triggered burst release and bacterial enrichment for enhanced tumor therapy. (A) FUS generates acoustic pressure fields, inducing both an acoustic radiation force gradient (from regions of high to low potential energy) and a forward-directed scattering force. These forces push bacteria away from the FUS focal zone. $w_{0}$ denotes the beam width at the focal point. (B and C) Simulation results showing the spatial distribution of acoustic pressure (B) and potential energy (C) calculated based on Gor’kov theory. The highest energy density is localized at the focal zone, enabling the directional control of bacterial positioning. (D) Conceptual workflow showing bacterial circulation, FUS-induced bacterial manipulation and sonopermeation, and enhanced therapeutic enrichment at the tumor site. (E) Mechanism of FUS-triggered burst release. FUS induces oscillation and collapse GVs within engineered bacteria, generating shock waves that lyse bacteria and release TRAIL.

In this study, we present a biohybrid living-material platform for FUS-triggered bacterial therapy that overcomes the limitations of gradual secretion and passive homing by acoustically pushing bacteria toward vessel walls and enriching burst-released therapeutics through sonopermeation. First, we used FUS to generate acoustic pressure fields that create localized potential energy (Fig. 1A to C). In a vascular context, these forces push bacteria toward vessel walls and enhance tumor permeability via sonopermeation, improving therapeutic accumulation (Fig. 1D). To enable burst release, we engineered *Escherichia coli* Nissle 1917 (EcN), a probiotic strain with a documented history of human administration and prior use in biomedical studies [17,18], to coexpress GVs and tumor necrosis factor-related apoptosis-inducing ligand (TRAIL), referred to as EcN-GVs + TRAIL. Upon FUS exposure, GVs collapse and generate shock waves that lyse bacteria and release TRAIL, as an antitumor protein payload [19–21] (Fig. 1E). TRAIL has been reported to exhibit relative stability under thermal and mechanical stimuli, making it a suitable therapeutic payload for ultrasound-based delivery strategies [19,22]. Using a single intravenous administration of EcN-GVs + TRAIL combined with a single FUS session with an exposure duration within 2 min, experimental validation in a murine tumor model demonstrated an approximately 40% reduction in tumor volume. Relying solely on the probiotic strain EcN and FUS without additional chemical agents, this approach offers a straightforward and clinically translatable strategy for biohybrid tumor therapy [23,24]. Collectively, this work establishes an acoustically driven in vivo delivery and enrichment strategy that leverages ultrasound-induced mechanical forces to achieve burst release and localized therapeutic accumulation while highlighting a physically programmable framework for living bacterial therapeutics. To our knowledge, this is the first demonstration that combining burst release and enrichment through FUS markedly improves therapeutic efficacy. This strategy can be readily extended to diverse therapeutic proteins or engineered bacterial systems, leveraging ultrasound physics to establish externally actuated and programmable living therapeutics.

## Materials and Methods

### EcN strain construction

*E. coli* Mach-T1 cells were utilized in cloning procedures, specifically employing experimental plasmids such as pET28a_T7_ARG, which were sourced from Addgene (RRID: Addgene: 106474). The TRAIL-wt sequence was replicated based on data from a previous study [25]. For the construction of pCDF_pHCE-TRAIL and pCDF_pHCE-TRAIL-*gfp*, the Gibson Assembly method (NEBuilder HiFi DNA, New England Biolabs, USA) was used to construct pCDF_pHCE-TRAIL and pCDF_pHCE-TRAIL-*gfp*. To construct pCDF_pHCE_TRAIL, fragments were amplified from TRAIL-wt and pCDFDuet plasmids using primers HCE-trail_R/F and Trail_his_R/F, respectively. The green fluorescent protein (GFP) fragment was amplified from the pACYC-*gfp* using primers GFP_F/R, and the vector fragment was amplified from the plasmid pCDF_pHCE_TRAIL using primers Trail_G_F/R to construct pCDF_pHCE-TRAIL-*gfp*. Additionally, blunt-end ligation facilitated by DNA ligase (DiaStar Quick DNA Ligase, Solgent, Korea) and the ARG_T5F/R primer pair was used to create the pET_ARG plasmid from pET28a_T7_ARG. The constructed plasmid was introduced into EcN cells, producing EcN-TRAIL, EcN-GVs + TRAIL-*gfp*, EcN-GVs, and EcN-GVs + TRAIL. The primers, strains, and plasmids used are listed in Tables S1 and S2.

### EcN culture condition

The constructed strains were cultivated in terrific broth (TB) medium (TB-modified powder, Sigma, USA) containing 8 ml/l (0.8% v/v) glycerol. The medium was supplemented with antibiotics, specifically kanamycin (50 mg/l) and streptomycin (50 mg/l). For the expression of GVs, cultures were initiated from seed cultures, diluted to a final OD_600_ (optical density at 600 nm) of 0.1 in fresh TB media, and incubated at 37 °C. When the OD_600_ reached 0.5 to 0.8, an appropriate amount of isopropyl β-d-1-thiogalactopyranoside (IPTG) was added. The cultures were incubated at 30 °C and shaken at 200 rpm for 24 h.

### Analytical methods for GVs

GV expression was evaluated by measuring buoyancy. *E. coli* (5 × 10^9^ colony-forming units/ml) were centrifuged at 300*g* for 15 min, and the number of cells suspended in the supernatant was measured using a spectrophotometer (SHIMADZU UV-1280, Shimadzu, Japan). The fold change was calculated by dividing the OD value after centrifugation by the OD value before centrifugation. In addition, flow cytometry was performed to analyze morphology-associated light-scatter properties of engineered EcN. Forward scatter and side scatter were analyzed to evaluate relative cell size and intracellular structural complexity, respectively.

### Analytical method for TRAIL

TRAIL expressions from the constructed strain EcN-GVs, EcN-TRAIL, and EcN-GVs + TRAIL were assessed using sodium dodecyl sulfate–polyacrylamide gel electrophoresis (SDS-PAGE). Cells from cultured samples were lysed by sonication. The resulting lysates were loaded onto 12% polyacrylamide gels for SDS-PAGE. PageRuler Prestained Protein Ladder (Thermo Fisher Scientific, MA, USA) was used as the protein size marker. Protein samples were loaded, and a constant voltage of 140 V was applied to facilitate protein migration through the gel. Then, the gel was visualized with Coomassie Blue staining. A relative comparison of TRAIL expression levels at different IPTG concentrations was performed by tagging TRAIL with GFP. Fluorescence was analyzed using a microplate reader (VICTOR3 1420 Multilabel Counter, PerkinElmer, Waltham, MA, USA). The concentration was quantified using an enzyme-linked immunosorbent assay (ELISA) kit (BMS2004, Invitrogen, MA, USA).

### Simulation of cavitation and acoustic radiation force

Cavitation simulation was conducted using Bubblesim, a MATLAB-based tool [26]. The simulation employed the Rayleigh–Plesset model under the assumption of isothermal conditions in the blood. A 3-MHz rectangular ultrasound signal was applied to the 30-cycle bursts. The initial radius of the GVs was set to 0.425 μm, with a shell thickness of 2.4 nm [27,28]. The Gor’kov equation was used to calculate the potential energy [29]. A negative potential energy gradient was applied to determine the acoustic radiation force acting on the bacteria. The size, density, and speed of sound for the bacteria were set to 0.001 mm, 1,012 kg/m^3^, and 1,524 m/s, respectively [30]. Ultrasound parameters included a 3-MHz FUS with a Gaussian distribution, and the simulation was calculated at the focal point.

### Fluorescence imaging of EcN lysis

Cultured EcN-GVs + TRAIL was spread on a solid lysogeny broth (LB) medium with 10 μM IPTG and incubated at 30 °C. To facilitate ultrasound delivery, phosphate-buffered saline (PBS) diluted with propidium iodide (PI; P3566, Invitrogen) to a concentration of 2.4 μg/ml was added. The colonies were observed using an inverted microscope (IX-73, Olympus, Center Valley, PA, USA), and fluorescence changes were recorded using a camera (ORCA-Flash4.0 V3, Hamamatsu Photonics, Shizuoka, Japan). The ultrasound transducer’s position was controlled using a 3-axis linear motor (OSMS20-85; Sigma Koki Co. Ltd., Tokyo, Japan).

### Ultrasound exposure and mechanical acoustic characterization

The acoustic pressure of the custom-designed transducer was measured using a hydrophone (NH0040, Precision Acoustics, Dorchester, UK), and the impulse response was obtained using a pulser receiver (DPR500, Imaginant Inc., Pittsford, NY, USA) (Fig. S1). Ultrasound was applied at a frequency of 3 MHz, pulse repetition frequency (PRF) of 1 kHz, and duty factor of 1%. Using the measured −6-dB lateral beamwidth [full width at half maximum (FWHM) ≈ 1 mm] and an impulse response-based axial extent (≈1.1 mm), the focal volume was approximated as ~0.58 mm^3^ assuming an ellipsoidal focal region.

The mechanical index (MI) was calculated from the peak negative pressure at the focus measured using a free-field calibrated needle hydrophone and the center frequency [31]. With a center frequency of 3 MHz and a focal peak negative pressure of 5 MPa, MI was 2.89 ($\mathrm{MI}=\frac{\text{Peak negative pressure}}{\sqrt{\text{Frequency}}}=\frac{5\;\mathrm{MPa}}{\sqrt{3\;\mathrm{MHz}}}=2.89$), serving as a descriptive parameter of a mechanically dominant exposure regime rather than a regulatory safety metric.

The spatial-peak pulse-average intensity (*I*_SPPA_) and spatial-peak temporal-average intensity (*I*_SPTA_) were also calculated [32]. Using the measured root-mean-square (RMS) pressure, a duty factor of 1%, and standard soft-tissue acoustic properties, *I*_SPPA_ was estimated to be approximately 812 W/cm^2^ ($\begin{array}{c} I_{\text{SPPA}}=\frac{p_{\mathrm{RMS}}^{2}}{\rho c}= \end{array}$$\begin{array}{c} \frac{{\left(3.536\;\mathrm{MPa}\right)}^{2}}{1,000\;\mathrm{kg}/m^{3}\cdot 1,540\;m/s}=812\;W/cm^{2} \end{array}$), corresponding to an *I*_SPTA_ of approximately 8.1 W/cm^2^.

### Thermal modeling of ultrasound exposure

To estimate the potential thermal effects under the applied ultrasound exposure conditions, temperature-rise modeling was performed based on the actual experimental parameters. Assuming linear acoustic absorption, the average volumetric heat generation rate at the focus was defined as $q=2\alpha I_{\text{SPTA}}$, where α is the acoustic absorption coefficient of tissue [33]. The corresponding local temperature rise was estimated as $\Delta T=\frac{2\alpha I_{\text{SPTA}}∆t}{\rho C}$ [34]. The volumetric heat capacity of soft tissue was assumed to be $\rho C\approx 3.5-3.8\times 10^{6}\;J/\left(m^{3}\cdot K\right)$, and the acoustic absorption coefficient was taken as 0.5 to 1.0 dB/cm/MHz [35,36]. An effective exposure duration of ∆t=0.4s was used to reflect the applied pulsed ultrasound strategy. Under these assumptions, the estimated local temperature rise was limited to $\Delta T\approx 0.29\;\text{to}\;0.64^{{}^{\circ}}C$, corresponding to a thermal index for soft tissue (TIS)-equivalent value of approximately 0.3 to 0.6.

### Vascular mimic setup

A syringe pump (New Era Pump Systems Inc., Farmingdale, NY, USA) was used to create the flow of EcN-*gfp* and EcN-GVs + TRAIL-*gfp*. A silicone tube with an inner diameter of 0.3 mm and an outer diameter of 0.64 mm was connected to the syringe and inserted into a Petri dish with a drilled hole. A beaker was placed at the end of the tube to collect the bacterial suspensions. Ultrasound was applied at a frequency of 3 MHz, acoustic pressure of 5 MPa, PRF of 1 kHz, and duty factor of 1%.

### Tumor cell culture and transplantation

The 4T1 breast cancer cells [American Type Culture Collection (ATCC), catalog no. CRL-2539] were cultured in RPMI 1640 (LM011-01, Welgene, Gyeongsangbuk-do, Republic of Korea) supplemented with 10% fetal bovine serum (FBS; 16000-044, GIBCO, Grand Island, NY, USA) and 1% antibiotic–antimycotic (15240-062, GIBCO). The cells were incubated in a CO_2_ incubator and subcultured every 2 to 3 d using trypsin–EDTA. Cells at over 80% confluence were harvested, washed with PBS, and resuspended in PBS at 10^6^ cells/ml.

All animal experiments were conducted in compliance with the procedures approved by the Institutional Animal Care and Use Committee (IACUC) of Pohang University of Science and Technology (POSTECH) (number: POSTECH-2022-0114-R1). All in vivo experiments used 4- to 6-week-old female BALB/c mice weighing approximately 20 g. The animals were randomly assigned to experimental groups, and outcome assessments were performed in a blind manner to minimize bias. A tailored breathing mask was used to attach the mice to an isoflurane anesthesia system (VIP3000 Veterinary Vaporizer, Midmark, USA) to ensure that the mice remained anesthetized with 2% isoflurane. To establish tumor models in mice, 10^6^ 4T1 cells suspended in 100 μl of PBS were injected subcutaneously into the flank of each mouse.

### Validation of therapeutic accumulation

EcN-GVs + TRAIL was labeled with 1,1′-dioctadecyl-3,3,3′,3′-tetramethylindotricarbocyanine Iodide (DiR; Thermo Fisher Scientific Inc., USA), and both in vivo and ex vivo fluorescence imaging were performed to monitor bacterial accumulation in tumors. A 60-μl DiR solution was incubated at 37 °C for 30 min. Seven days after tumor implantation, 0.1 ml of DiR-labeled EcN-GVs + TRAIL (10^8^ cells/ml) was intravenously injected into the mice, followed by a 2-min ultrasound application to the tumor tissue. In a separate in vivo permeability experiment (Fig. S2), FUS was applied 2 d after injection, and tumor fluorescence was measured before and 30 min after exposure. The 2-min exposure defined a circulation-dependent interaction window in vivo. The tumor was visible, allowing manual transducer positioning under visual guidance. The focus was aligned with the tumor depth and raster-scanned along the *x* and *y* axes to ensure full coverage. Two days after injection, mice were sacrificed, and tumor tissues were excised for imaging and assessment of inflammatory responses. In addition, major organs, including the heart, lung, liver, spleen, and kidney, were dissected without perfusion for ex vivo fluorescence imaging. An IVIS imaging system (IVIS Lumina II, Caliper Life Sciences, Hopkinton, MA, USA) was used to visualize the drug delivery. Photographic and luminescent images were acquired using excitation and emission filters of 740 and 790 nm, respectively. Fluorescence intensity was quantified by applying uniform regions of interest (ROIs) to each organ, following the manufacturer’s recommended analysis guidelines.

Reverse transcription quantitative polymerase chain reaction (RT-qPCR) was performed to assess inflammatory responses. DNA was extracted using the Quick-DNA Miniprep Plus Kit (Zymo Research, Irvine, CA, USA). Fluorescent signals were obtained using TaqMan probes, primers [interleukin-1α (IL-1α), IL-1β, and TNF-α], polymerase, dNTPs, and buffer. Relative expression levels were calculated using the ΔΔCt method and standardized against the housekeeping gene (18s). Immunofluorescence (IF) staining was performed to visualize the inflammatory responses. The excised tissues were fixed on slides, and imaging was conducted for 4′,6-diamidino-2-phenylindole (DAPI), TRAIL, and TNF-α at 405-, 488-, and 594-nm wavelengths, respectively.

### Validation of tumor growth inhibition

The therapeutic antitumor effects were evaluated using a mouse tumor model. Seven days after tumor implantation, when the tumors had grown to approximately 50 mm^3^, the mice were randomly divided into 4 groups (control, only injection, only FUS, and injection + FUS). Tumor volume measurements and image analyses were performed in a blinded manner, with investigators unaware of treatment group allocation. In the injection and injection + FUS groups, 0.1 ml of DiR-labeled EcN-GVs + TRAIL (10^8^ cells/ml) was intravenously injected into the mice. Additionally, ultrasound was applied only to the FUS and injection + FUS groups. Ultrasound was applied at a frequency of 3 MHz, acoustic pressure of 5 MPa, PRF of 1 kHz, and duty factor of 1%. The tumor volume and body weight of the mice were measured every 2 d. Images of the tumors in the mice were obtained at 4-d intervals. Tumor volume was calculated using the formula $V=\left(a\times b^{2}\right)/2\;$, where *a* is the longest radius and *b* is the shortest radius. Statistical significance was determined by 1- or 2-way analysis of variance (ANOVA) (*P* < 0.05). After 21 d, the tumor-bearing mice were sacrificed, and their organs and tumors were dissected and fixed in 10% neutral buffered formalin (NBF). The tumor and organ sections were stained with hematoxylin and eosin (H&E) for histological analysis.

### Statistical analysis and visualization

Statistical analysis was performed using Prism software (GraphPad Software Inc., San Diego, CA, USA). Each graph represents the mean ± standard deviation. Results were considered significant when *P < 0.05, **P < 0.01, ***P < 0.001, and ****P < 0.0001. Graphs and illustrations were generated using Prism and MATLAB (MathWorks, Natick, MA, USA), and BioRender. Images were analyzed using ImageJ, MetaMorph (Molecular Devices LLC, San Jose, CA, USA), and Living Image software (Revvity, Hopkinton, MA, USA).

## Results

### Coexpression of GVs and TRAIL

**Fig. 2.**
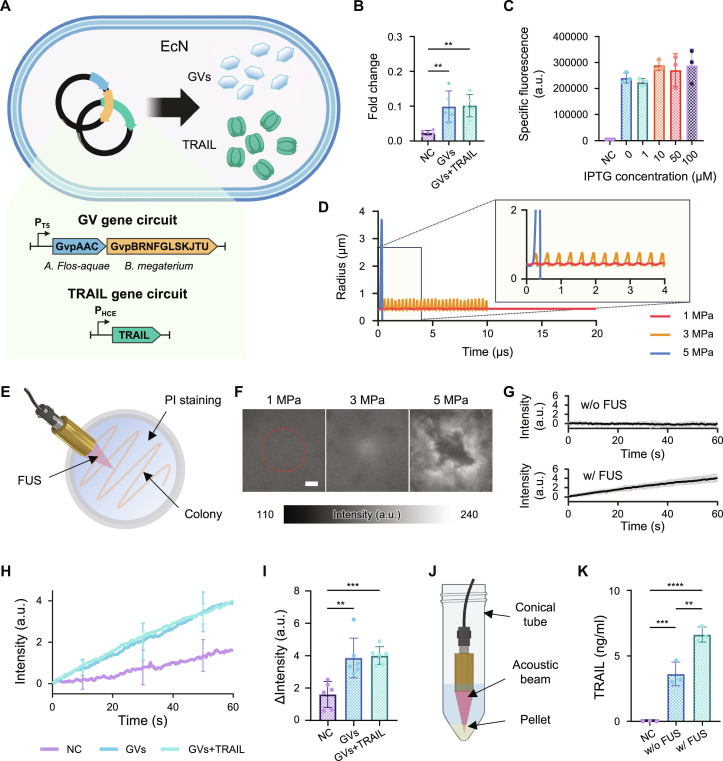
FUS-triggered lysis of EcN and release of TRAIL. (A) Schematic representation of EcN-GVs + TRAIL construction. (B) Fold change of GV-expressing strains. It was calculated as the ratio of OD_600_ of the supernatant to that of the total solution. Data are presented as mean ± SD (*n* = 5), which were statistically analyzed using one-way analysis of variance (ANOVA) tests. **P < 0.01. (C) Specific fluorescence measurement of EcN-GVs + TRAIL-gfp under varying IPTG concentration, demonstrating unaffected TRAIL expression regardless of GV induction. Data are presented as mean ± SD (*n* = 3). (D) Simulation of GV size dynamics under different acoustic pressures, predicting collapse at ≥5 MPa. (E) Schematic of the experimental setup for visualizing lysis of EcN colonies. Bacterial colonies stained with PI to visualize lysis. (F) Lysis behavior observed under increasing acoustic pressures (1, 3, and 5 MPa). The red dotted circle indicates the focal area of the FUS. Scale bar, 100 μm. (G) Time-dependent fluorescence intensity changes with and without FUS. The gray lines represent the results of each experiment, while the black line indicates the average of the experiments (*n* = 6). (H) Comparison of lysis dynamics in EcN, EcN-GVs, and EcN-GVs + TRAIL strain. Data are presented as mean ± SD (*n* = 6). (I) Quantification of fluorescence intensity before and after FUS exposure. Data are presented as mean ± SD (*n* = 6), which were statistically analyzed using one-way ANOVA test. **P < 0.01, ***P < 0.001. (J) Schematic of TRAIL release quantification experiment after centrifugation and FUS application. (K) ELISA results for quantification of TRAIL detection. Data are presented as mean ± SD (*n* = 3), which were statistically analyzed using one-way ANOVA test. **P < 0.01, ***P < 0.001, ****P < 0.0001. In (B), (H), and (I), NC, GVs, and GVs + TRAIL refer to the EcN, EcN-GVs, and EcN-GVs + TRAIL strains, respectively. In (C), NC refers to the EcN-gfp. In (K), NC refers to the EcN-GVs strain.

We developed a bacteria-based, ultrasound-responsive tumor therapy system by coexpressing GVs and TRAIL (Fig. 2A). Previously studied acoustic reporter systems were introduced into the EcN for GV expression (EcN-GVs) [37]. To optimize GV expression, we tested various concentrations of IPTG to determine the most appropriate level for optimal GV expression (Fig. S3A). GV expression was indirectly measured by centrifuging the samples and measuring the OD_600_ values of floating cells in the supernatant. GV expression increased with IPTG concentration and was sufficient at 10 μM. Since higher IPTG levels could burden cell physiology, 10 μM was selected as the standard concentration for further experiments.

Next, we examined whether coexpression of TRAIL affects GV expression. For the coexpression of GVs and TRAIL, the plasmid pCDF_pHCE-TRAIL was introduced into EcN-GVs. By comparing the fold changes in OD_600_ values between the total solution and the supernatant across different experimental groups, we confirmed that TRAIL expression had no impact on GV expression (Fig. 2B). Additionally, TRAIL was expressed in both the TRAIL-only (EcN-TRAIL) and EcN-GVs + TRAIL strains, indicating no effect of GV expression on TRAIL expression (Fig. S3B). To quantitatively assess whether coexpression with GVs affects TRAIL expression, we fused GFP to TRAIL (EcN-GVs + TRAIL-*gfp*) and compared the relative expression levels by measuring the fluorescence across varying IPTG concentrations (Fig. 2C). The results showed that TRAIL expression, regulated by the constitutive promoter, was unaffected by IPTG concentration, confirming that the expression of TRAIL and GVs did not interfere.

### EcN lysis and TRAIL release

Extending previous concepts of GV-mediated bacterial lysis for mechanotherapy, we employed ultrasound to induce burst release of therapeutic proteins from EcN [12,13]. Ultrasound-driven compression and expansion of GVs lead to collapse of GVs, generating shock waves that trigger EcN lysis. Considering the physical properties of the GVs, we simulated changes in their radius over time under varying acoustic pressures. The results showed that greater pressure induced more marked size changes, whereas excessive pressure resulted in nonlinear responses (Fig. 2D). The simulations revealed that GV collapse occurred at an acoustic pressure of 5 MPa, whereas only oscillations were observed at pressures below 3 MPa.

To experimentally validate these findings, we added PI diluted in PBS to EcN colonies and observed lysis by fluorescence (Fig. 2E). Colonies were used to ensure that bacteria remained localized during ultrasound exposure. The acoustic focus was positioned at the center of the recording field, and lysis dynamics were monitored under varying acoustic pressures (Fig. 2F). No fluorescence changes were observed at 1 MPa, while a slight increase in fluorescence was detected at 3 MPa. At the predicted collapse pressure of 5 MPa, fluorescence intensity increased sharply upon the onset of ultrasound application, pushing out visible colonies. These results demonstrated that a pressure of 5 MPa was sufficient to induce EcN lysis and enable bacterial manipulation. With this pressure level, we observed fluorescence changes over time in colonies exposed to FUS and in those that were not (Fig. 2G). No fluorescence changes were observed without FUS, indicating that lysis did not occur. In contrast, fluorescence increased when 5-MPa FUS was applied, supporting that lysis had occurred.

To verify whether the lysis effect of the EcN was specifically due to GVs, we compared the EcN, EcN-GVs, and EcN-GVs + TRAIL strains (Fig. 2H). When FUS at 5 MPa was applied to the EcNs [negative control (NC)], the fluorescence increased only slightly, suggesting minimal lysis. In contrast, both EcN-GVs and EcN-GVs + TRAIL showed a sharp increase in fluorescence intensity, indicating active lysis. Comparison of fluorescence values before and after FUS exposure revealed minimal lysis in EcN, whereas the EcN-GVs and EcN-GVs + TRAIL groups exhibited significant lysis (Fig. 2I). No significant differences were observed between these 2 groups, suggesting that coexpression of TRAIL did not impede EcN lysis. Together, these results demonstrate that bacterial lysis under FUS occurs specifically in GV-expressing strains at 5 MPa, independent of TRAIL coexpression.

We also evaluated the release of drugs facilitated by lysis. The EcN-GVs + TRAIL strains were pelleted by centrifugation, followed by FUS application, and the amount of TRAIL released was quantified (Fig. 2J). Pelleting concentrated the suspended bacteria and ensured that the FUS energy was directed more effectively toward the cells. FUS could not be evenly applied to all bacteria; nevertheless, more TRAIL was released from ultrasound-treated samples (Fig. 2K). This approach demonstrated that TRAIL burst release was amplified by bacterial lysis. Because ELISA relies on epitope-specific antibody recognition, the preserved ELISA signal under FUS conditions suggests that the released TRAIL retained an antibody-recognizable conformation. In addition, flow cytometry analysis showed that GV expression increased side scatter without substantial changes in forward scatter, while FUS exposure did not induce additional detectable shifts in these parameters in intact bacterial populations (Fig. S4).

### Manipulation and sonopermeation for therapeutic enrichment

**Fig. 3.**
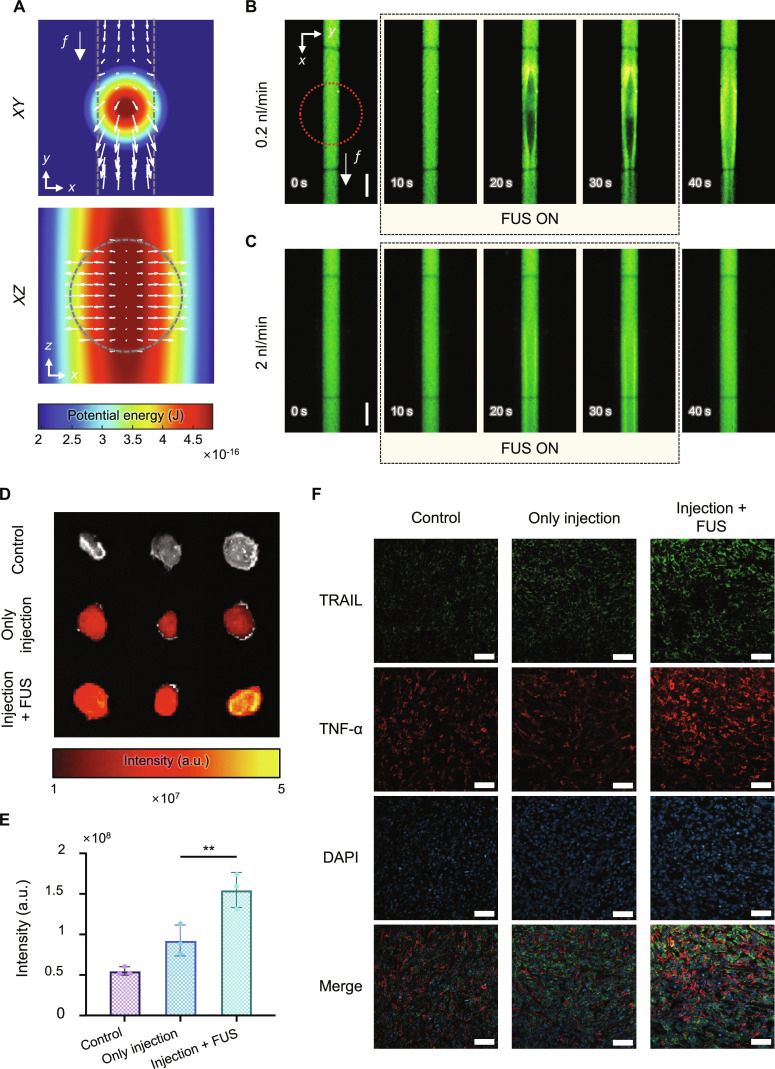
FUS-triggered therapeutic enrichment through acoustic manipulation and sonopermeation. (A) Simulation of the acoustic radiation force showing potential energy distribution. Arrows indicate force vector directions acting on bacterial cells. (B and C) Fluorescence images showing the manipulation of EcN-GVs + TRAIL-gfp under FUS at flow rates of 0.2 (B) and 2 nl/min (C). *f* refers to the fluid flow, and the bacteria move in the direction of the arrow. The red dotted circle indicates the focal area of the FUS. Scale bar, 400 μm. (D) Fluorescence imaging of excised tumor tissues demonstrating enhanced therapeutic accumulation (*n* = 3). (E) Quantification analysis of fluorescence intensity. Data are presented as mean ± SD (*n* = 3), which were statistically analyzed using one-way ANOVA test. **P < 0.01. (F) Images of tumor sections showing TRAIL (green), TNF-α (red), and DAPI (blue). Scale bars, 50 μm.

Ultrasound can manipulate bacteria by trapping them with acoustic tweezers to achieve local enrichment, although the effect is typically limited to a few vessels [16]. In the case of single-element FUS, trapping is challenging; however, bacteria can still be manipulated by the net radiation force, enabling broader influence. As schematically illustrated in Fig. 1A, the scattering force aligns with the direction of ultrasound propagation, whereas the gradient force arises from spatial variations in potential energy. Upon ultrasound application, bacteria experience a force directed away from the ultrasound focus. A numerical simulation of acoustic radiation force showed that ultrasound disrupted bacterial trajectories and pushed them toward the vascular walls (Fig. 3A). This facilitates the enrichment of bacteria along the vessel wall and their retention at the target site.

To validate our simulation, we developed a vascular mimic system to observe the effect of FUS on bacteria within fluid flow (Fig. S5A). We used the same ultrasound conditions for lysis, employing EcN-GVs + TRAIL and EcN-GVs + TRAIL-*gfp*. The flow rate was set at 0.2 and 2 nl/min, comparable to the capillary flow rate in mice in the nl/min range [38]. As a result, FUS interfered with bacterial movement, leading to accumulation outside the focal point of the ultrasound application (Fig. 3B and Fig. S5B). At a low flow rate of 0.2 nl/min, the slow fluid flow allowed FUS to disrupt movement almost entirely within the focal region, resulting in bacterial migration primarily along the vessel walls. At a higher flow rate of 2 nl/min, although FUS could not interrupt the flow, it still pushed bacteria toward the vessel walls (Fig. 3C and Fig. S5C). In addition, we confirmed a similar trend in GV-negative bacteria, indicating that GV expression had minimal impact on manipulation in the vascular mimicry system (Fig. S6). These results suggest that FUS pushes bacteria toward the vessel walls, which could increase their chance of extravasation into tumor tissue.

Next, we evaluated whether FUS could enhance therapeutic enrichment. EcN-GVs + TRAIL was intravenously injected into mice, and the effects of FUS treatment were compared. Tumor tissues were excised, and DiR fluorescence was measured, with its intensity serving as an indicator of therapeutic accumulation (Fig. 3D and E and Fig. S7). The higher fluorescence intensity observed in the FUS-treated group suggests enhanced therapeutic accumulation resulting from both bacterial manipulation and sonopermeation effects. Bacterial manipulation, mediated by acoustic radiation forces, pushed bacteria toward the vessel walls, facilitating their retention at the target site, while sonopermeation increased tumor permeability, enabling greater penetration of bacteria and released therapeutics into tumor tissues. Although it is difficult to quantitatively assess the individual contribution of each effect, these results demonstrate that FUS enhances therapeutic enrichment through the synergistic action of mechanical manipulation and permeability enhancement. Consistent with this interpretation, an independent in vivo permeability experiment demonstrated a significant ultrasound-induced increase in tumor fluorescence after FUS application, providing in vivo evidence supportive of permeability modulation (Fig. S2).

We evaluated whether this accumulation could enhance bacterial and TRAIL delivery. Compared to the group relying solely on the intrinsic targeting ability of the injected bacteria, the FUS-treated group exhibited approximately a 3-fold increase in IL-1α and IL-1β (Fig. S8). TNF-α also increased by about 2-fold, albeit with higher variability. The enhanced inflammatory response also indicates that bacterial accumulation at the tumor site was effectively increased by FUS. Furthermore, fluorescence imaging showed increased TRAIL delivery and TNF-α expression after FUS treatment (Fig. 3F), consistent with the presence of antibody-recognizable TRAIL within the tumor microenvironment. Nonspecific fluorescence signals were assessed to confirm that each antibody specifically reflected the target (Fig. S9). Collectively, these findings suggest that FUS amplifies therapeutic accumulation by promoting bacterial enrichment and TRAIL delivery.

### Antitumor efficacy of FUS

**Fig. 4.**
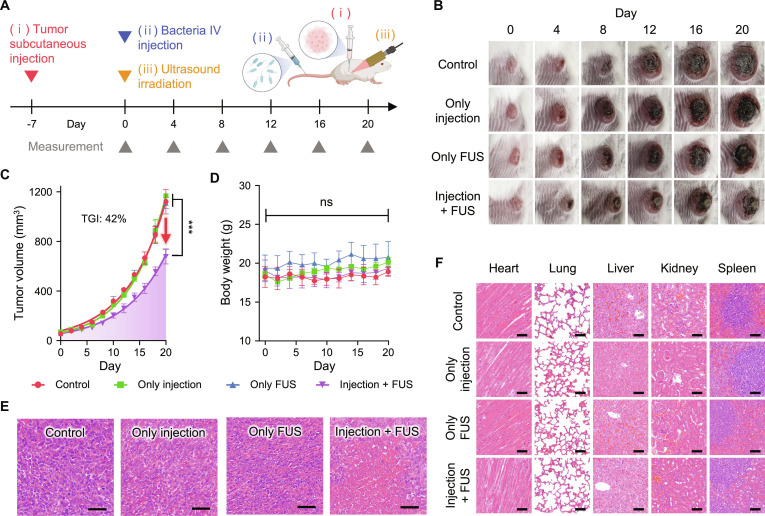
In vivo antitumor efficacy of FUS-triggered bacteria therapy. (A) Experimental timeline. 4T1 breast cancer cells were implanted in mice to establish tumors, followed by intravenous injection of EcN-GVs + TRAIL and application of FUS treatment. (B) Representative images of tumor-bearing mice across different treatment groups: control, EcN-GVs + TRAIL only, FUS only, and combined EcN-GVs + TRAIL with FUS. (C) Tumor growth curves demonstrating that only the combination of EcN-GVs + TRAIL and FUS significantly inhibited tumor progression compared to all other groups. (D) Body-weight monitoring indicates no systemic toxicity. (E) H&E staining of tumor tissues confirming therapeutic efficacy. (F) H&E analysis of major organs showing no observable tissue damage. In (C) and (D), data are presented as mean ± SD (*n* = 3), which were statistically analyzed using one-way ANOVA test. ***P < 0.001. The scale bars in (E) and (F) represent 50 μm.

The tumor therapy efficacy was validated using a 4T1-bearing mouse allograft tumor model. 4T1 breast cancer cells were implanted 7 d before the experiment. On day 0, EcN-GVs + TRAIL was intravenously injected, followed by FUS treatment (Fig. 4A). Tumor progression was monitored every 2 d for tumor volume and body weight, while images of tumor-bearing mice were captured every 4 d. The experimental groups were as follows: EcN-GVs + TRAIL alone, FUS alone, and a combination of EcN-GVs + TRAIL and FUS. In this experiment, tumor growth was inhibited within a 2-min ultrasound application following bacterial injection, achieving a tumor growth inhibition (TGI) of 42% (Fig. 4B and C). To complement this group-level efficacy, individual-level tumor progression and conservative summary analyses were performed to capture treatment-dependent disease dynamics across animals (Fig. S10). We found that no therapeutic effects were observed in groups that received injections or FUS only. These results suggest that neither the intrinsic targeting and therapeutic effects of bacteria nor the application of FUS alone was sufficient for effective tumor suppression. In contrast, the proposed strategy, injection + FUS group, contributed synergistically to the observed therapeutic efficacy. Consistent with these findings, ex vivo fluorescence imaging revealed pronounced tumor-associated signals predominantly in the FUS-treated group (Fig. S11 and Table S3).

Additionally, no significant changes in body weight were observed, indicating that the treatment did not induce systemic toxicity (Fig. 4D). H&E staining of the tumor tissue confirmed marked cancer cell damage and therapeutic efficacy in the injection + FUS group (Fig. 4E). Furthermore, no appreciable tissue damage was observed in nontumor tissues (Fig. 4F). These results demonstrate that FUS enables rapid and noninvasive tumor suppression without systemic toxicity.

## Discussion

In addition to achieving burst release and enrichment, this study addresses key biomedical engineering criteria and practical constraints relevant to clinical translation. Previous approaches typically required more than 10 min of ultrasound exposure, often repeated over several days with carefully controlled timing [9,14–16]. In contrast, our approach achieved therapeutic effects with a single 2-min FUS treatment administered immediately after bacterial injection. Several representative studies rely on laboratory strains such as BL21 or MG1655 [9,13–16], whereas we employed EcN, a probiotic strain widely studied and used in humans. In addition, our platform does not rely on external agents such as nanosonosensitizers or immune modulators [12–15]. Unlike acoustic tweezers, our method is simple and enables manipulation across multiple vessels and, for the first time, utilizes bacterial lysis to achieve both drug release and therapeutic effect [12,16]. These features highlight the clinical potential of our approach by combining procedural simplicity, biological safety, and therapeutic efficacy.

This study also contributes to ultrasound engineering by demonstrating a FUS approach that induces bacterial lysis using MHz-frequency ultrasound, overcoming the limitation that cavitation is efficiently induced only at kilohertz frequencies with low spatial resolution [39,40]. Moreover, enhanced membrane permeability, a mechanism widely applied for drug delivery across the blood–brain barrier (BBB) [41–44], has not been explored in bacterial therapy. This work expands the paradigm of ultrasound-based mechanotherapy, bridging MHz-frequency precision with bacterial control and drug delivery applications.

Despite these advantages, intravenous administration of live bacteria inherently requires careful consideration of systemic safety, including off-target colonization, bacterial persistence, and post-treatment controllability [4]. From this perspective, the present platform is structurally compatible with established bacterial containment strategies: EcN remains sensitive to commonly used antibiotics, enabling pharmacological clearance if required, and synthetic biology-based kill-switch designs reported in prior studies could be readily integrated in future iterations [3,23]. Notably, the FUS-triggered lysis strategy employed here may also contribute to spatial controllability, as localized ultrasound exposure effectively disrupts bacterial viability.

The present study focuses on an integrated, acoustically controlled in vivo delivery and enrichment process rather than isolating individual contributing mechanisms, with more targeted experiments needed to further extend this framework. One major challenge is the real-time tracking of bacterial movement within the microvasculature in vivo [45]. Flap models have been used to visualize bacterial behavior, but they do not fully replicate the noninvasive condition of a living mouse [16,46]. Second, future studies should aim to disentangle overlapping mechanisms, including immune modulation, apoptosis, sonopermeation, and bacterial enrichment, to enable more precise mechanistic refinement. In particular, ultrasound-mediated immunotherapy may provide synergistic benefits, as ultrasound can modulate the immune response by polarizing macrophages or converting immunologically cold tumors into hot tumors [5,47,48]. Third, while the structural integrity of TRAIL after FUS exposure was supported by preserved antibody recognition and prior reports of thermal and mechanical stability [19,22], additional analysis is required to determine the biological activity of TRAIL released from lysed bacteria under this acute delivery scheme.

For rigorous evaluation of in vivo efficacy and translational relevance, additional analyses aimed at strengthening stability, systemic characterization, and long-term outcome assessment are ultimately required. These include plasmid retention or expression stability analyses, quantitative biodistribution and clearance profiling of intravenously administered bacteria, and statistically powered survival analyses to determine whether transient tumor growth suppression translates into improved overall survival [4]. Although these analyses were beyond the scope of the present pilot-scale in vivo study, independent validation in adequately powered cohorts will be essential to confirm the robustness and generalizability of the observed therapeutic effects. Addressing these aspects in future studies is expected to further strengthen and extend the present integrated in vivo delivery framework.

In summary, we have developed a FUS-controlled bacterial therapy platform that enables both burst release of therapeutic proteins and localized enrichment at tumor sites. By engineering EcN to coexpress GVs and TRAIL, we achieved a substantial therapeutic effect in a murine tumor model using only a single 2-min FUS exposure and no auxiliary agents. This strategy provides a controllable, efficient, and clinically translatable approach for bacteria-based tumor therapy and can be broadly applied to other therapeutic payloads and disease models.

## Data Availability

The datasets used and/or analyzed during the current study are available from the corresponding authors on reasonable request.
