# Supplementary material for: Focused Ultrasound-Triggered Burst Release and Enrichment of Engineered Bacteria for Tumor Therapy
Source: Biomater Res. 2026 Apr 16;30:0354. doi: 10.34133/bmr.0354 (PMC13084060; doi:10.34133/bmr.0354)
Supplement: Supplementary 1 — Figs. S1 to S11 Tables S1 to S3 [file bmr.0354.f1.docx]

**Supplementary Materials**

Title

Focused Ultrasound-Triggered Burst Release and Enrichment of Engineered Bacteria for Tumor Therapy

**Authors**

Jinhee Yoo^1, †^, Yunhee Hwang^2, †^, Mihyeon Park^3, †^, Honghyeon Ha^4^, Myeong Ryeol Choi^5^, Sung In Lim^6^, Byullee Park^7^, Yong Joo Ahn^5^, Won Jong Kim^3, *^, Gyoo Yeol Jung^2, *^, and Hyung Ham Kim^1, 4, *^

**Affiliations**

^1^ Department of Electrical Engineering, Pohang University of Science and Technology, Pohang 37673, Republic of Korea.

^2^ Department of Chemical Engineering, Pohang University of Science and Technology, Pohang 37673, Republic of Korea

^3^ Department of Chemistry, Pohang University of Science and Technology, Pohang 37673, Republic of Korea

^4^ Department IT Convergence, Pohang University of Science and Technology, Pohang 37673, Republic of Korea

^5^ Medical Science and Engineering, Pohang University of Science and Technology, Pohang 37673, Republic of Korea

^6^ Department of Chemical Engineering, Pukyong National University, Busan 48513, Republic of Korea.

^7^ Department of Biophysics, Institute of Quantum Biophysics, Sungkyunkwan University, Suwon 16419, Republic of Korea.

^†^ These authors contributed equally to this work

^*^ Address correspondence to: Won Jong Kim; [wjkim@postech.ac.kr](mailto:wjkim@postech.ac.kr), Gyoo Yeol Jung; [gyjung@postech.ac.kr](mailto:gyjung@postech.ac.kr), and Hyung Ham Kim; [david.kim@postech.ac.kr](mailto:david.kim@postech.ac.kr)

**Supplementary Figures**

**
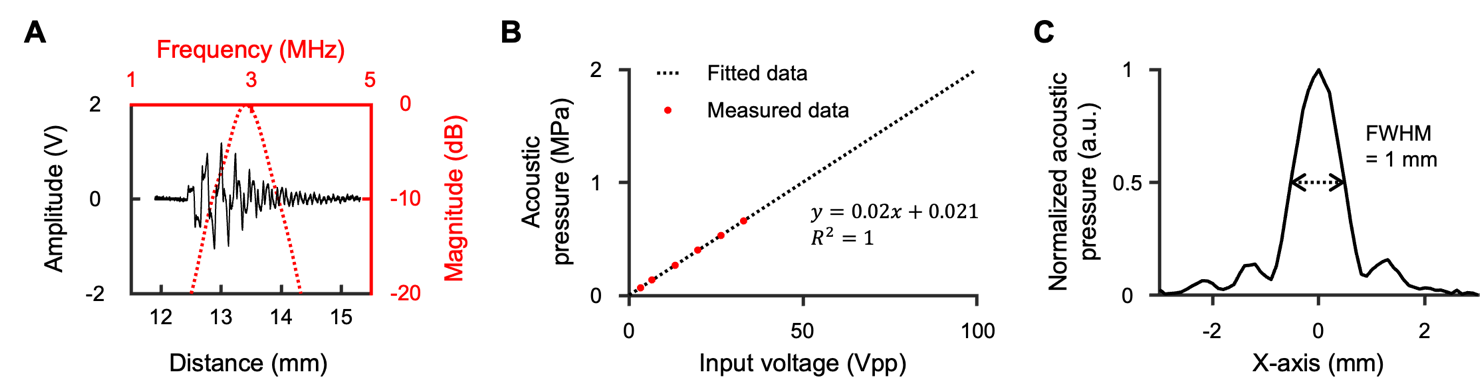
**

**Fig. S1.** Characterization of the custom-designed ultrasound transducer. (A) Impulse response profile of the transducer. (B) Relationship between acoustic pressure and input voltage. (C) Lateral beam width distribution as measured by acoustic pressure mapping, confirming focused ultrasound delivery efficiency. Using the measured −6 dB lateral beamwidth (FWHM ≈ 1 mm) and an impulse-response–based axial extent (≈ 1.1 mm), the focal volume was approximated as ~0.58 mm³ assuming an ellipsoidal focal region.

**
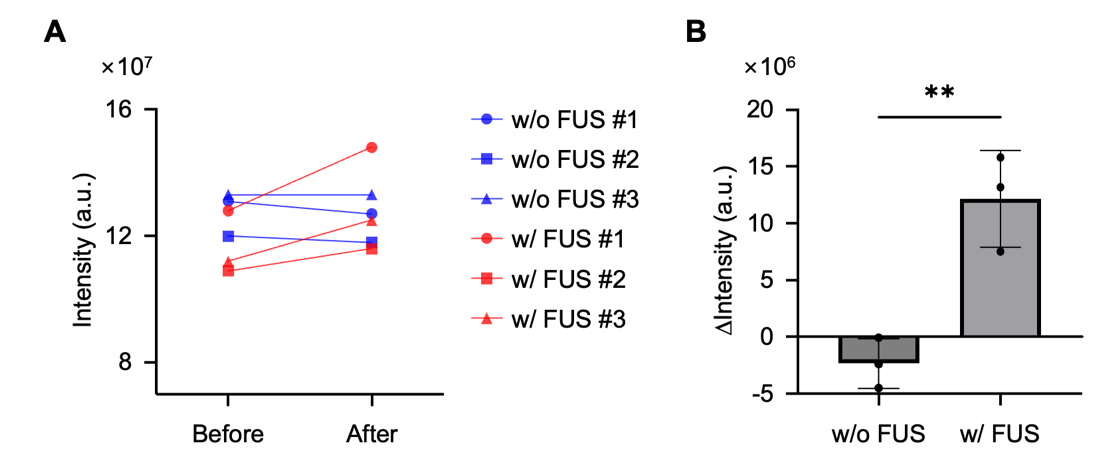
**

**Fig. S2.** In vivo ultrasound-induced modulation of tumor permeability. (A) Changes in tumor fluorescence intensity measured in vivo before and 30 min after localized FUS application, performed two days after intravenous injection of DiR-labeled EcN-GVs+TRAIL. Each line represents an individual mouse, with measurements obtained under without FUS (w/o FUS) and with FUS (w/ FUS) conditions. (B) Quantification of fluorescence intensity change (ΔIntensity) comparing tumors without and with FUS treatment. Data are presented as mean ± SD (n = 3), which were statistically analyzed using an unpaired two-tailed Student’s t-test. **p < 0.01.

**
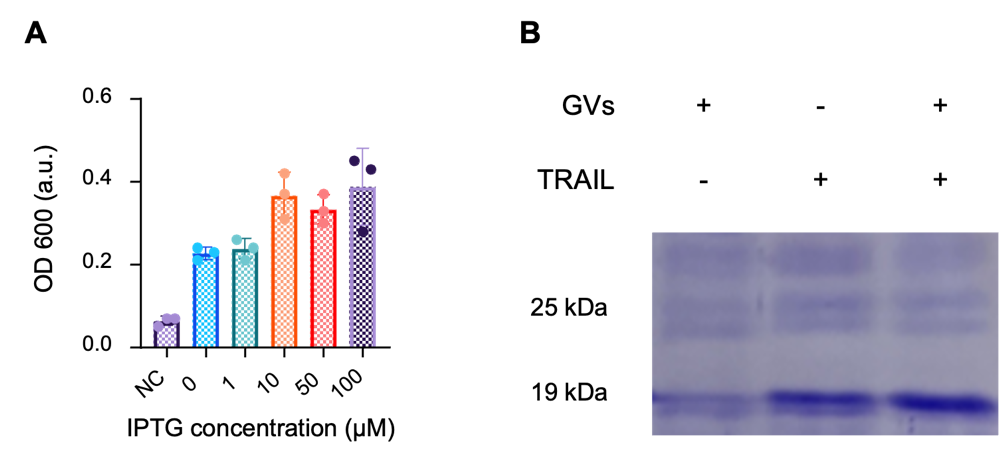
**

**Fig. S3.** Optimization of engineered bacteria expressing GVs and TRAIL. (A) Quantification of GV expression by measuring OD600 of supernatant at different concentrations of IPTG. NC refers to the EcN strain. Data are presented as mean ± SD (n = 3). (B) SDS-PAGE analysis confirming TRAIL expression in the EcN-GVs, EcN-TRAIL, and EcN-GVs+TRAIL strain.

**
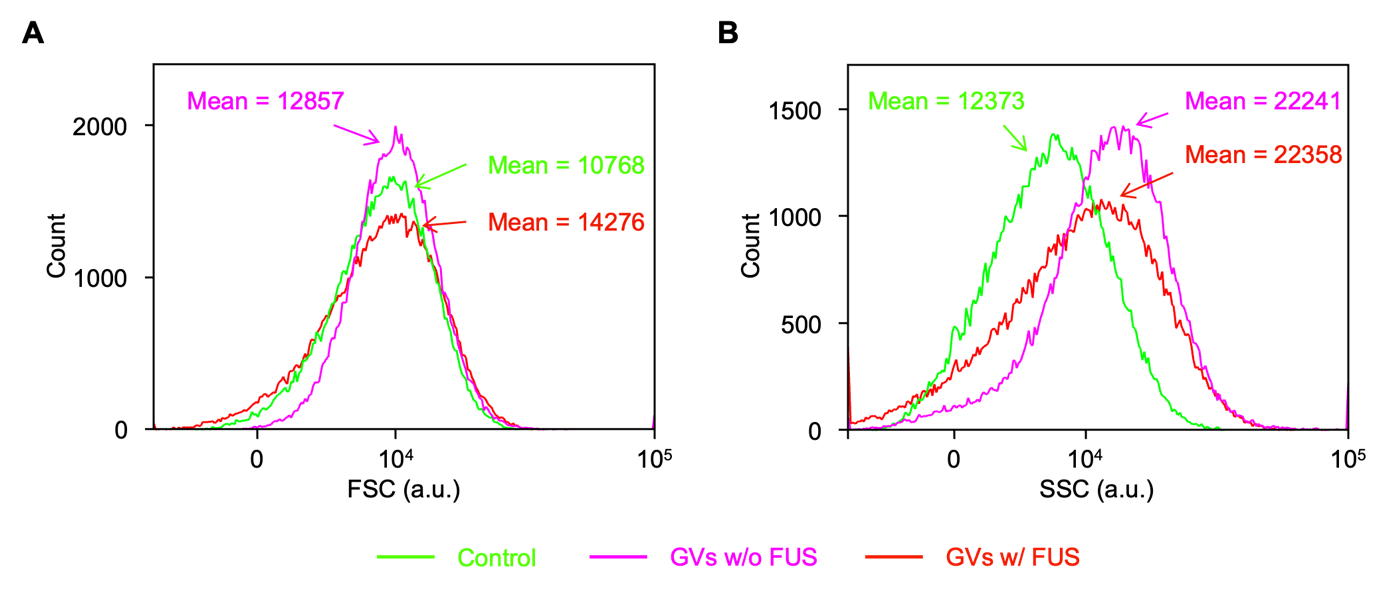
**

**Fig. S4.** Flow cytometry analysis of morphology-related scatter properties of engineered EcN. (A) Forward scatter (FSC) distributions of control EcN, GV-expressing EcN without FUS, and GV-expressing EcN after FUS exposure. FSC values showed only modest differences among groups, indicating no substantial change in overall cell size. (B) Side scatter (SSC) distributions of the same groups. GV-expressing EcN exhibited a pronounced rightward shift in SSC compared to control cells, whereas SSC values remained comparable before and after FUS exposure, indicating increased intracellular scattering associated with GV expression without additional effects from FUS.

**
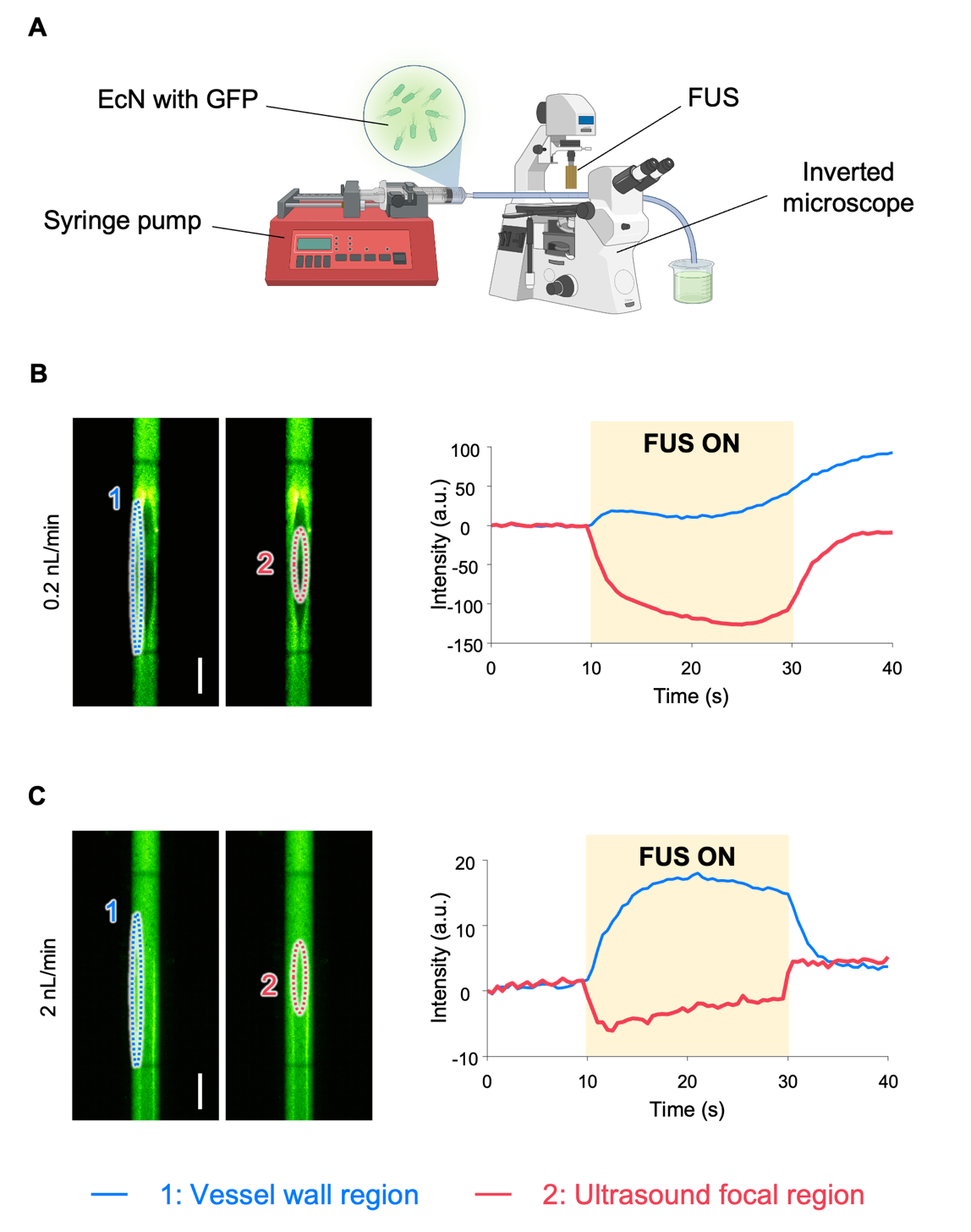
**

**Fig. S5.** Experimental setup of a vascular mimic system to observe bacterial dynamics under flow. (A) Setup and results of the vessel-mimicking system. (B, C) Changes in bacterial localization in the vessel wall region (1, blue) and the ultrasound focal region (2, red) at flow rates of 0.2 and 2 nL/min, respectively. Upon FUS application, bacterial density decreases in the ultrasound focal region and increases near the vessel wall in both cases. In (B), the increase in vessel wall intensity after FUS is turned off indicates that previously blocked bacteria flowed downstream. The scale bars represent 400 μm.

**
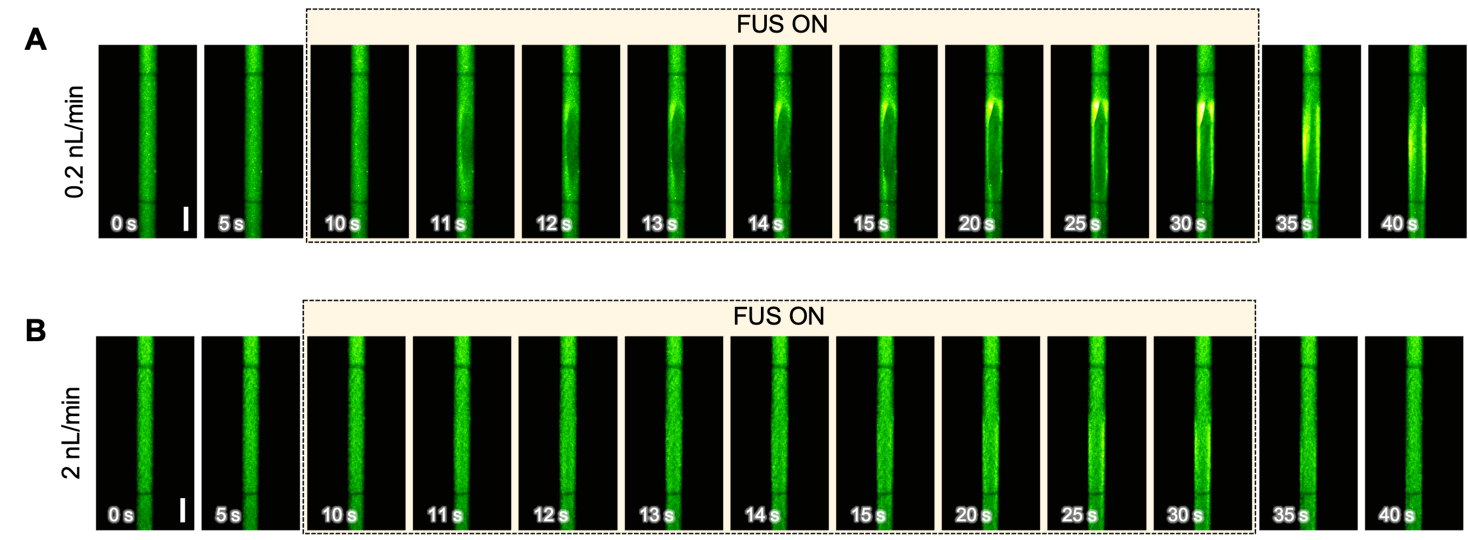
**

**Fig. S6.** Time-lapse analysis of EcN-gfp movement in a vascular mimic environment under FUS exposure. Flow rates of (A) 0.2 nL/min and (B) 2 nL/min were applied. FUS application influenced bacterial trajectories, promoting accumulation along vessel walls regardless of flow rate and GV expression.


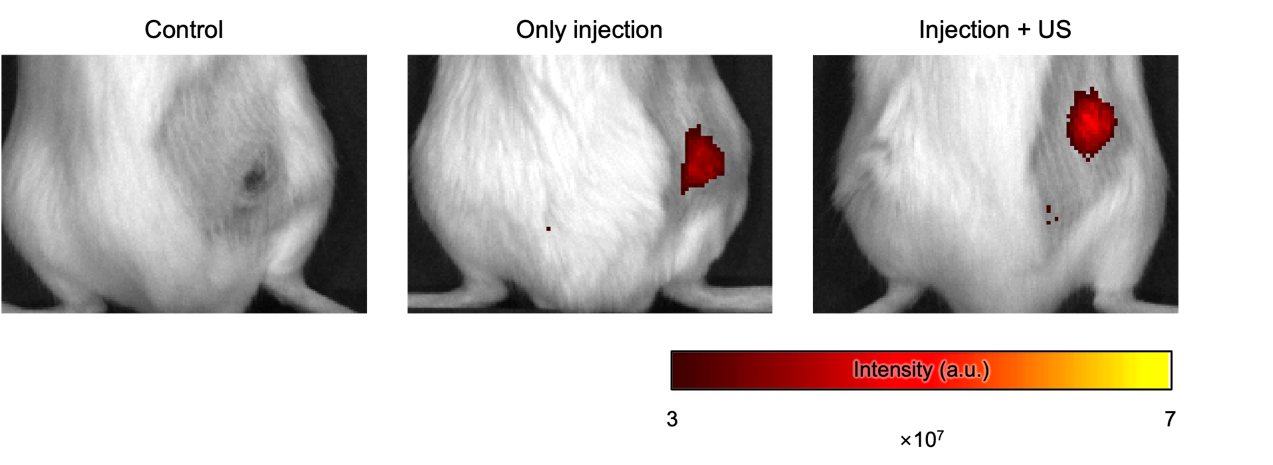


**Fig. S7.** In vivo fluorescence imaging of tumors before excision. Representative fluorescence images of tumor-bearing mice under each experimental condition, captured before tumor harvesting, demonstrating enhanced bacterial accumulation and drug delivery.


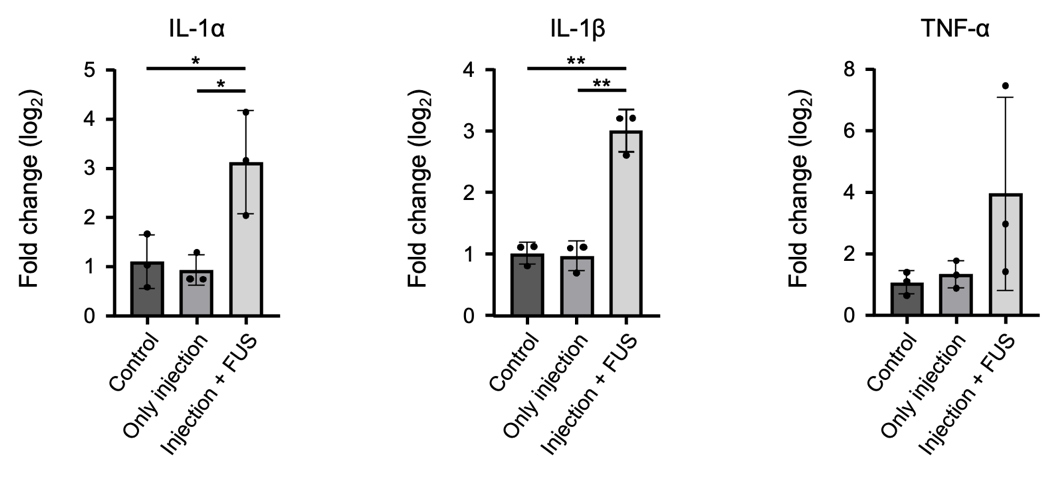


**Fig. S8.** Analysis of inflammatory markers (IL-1α, IL-1β, TNF-α) showing increased expression following FUS treatment. These data are presented as mean ± SD (n = 3), which were statistically analyzed using one-way ANOVA tests. *p < 0.05, **p < 0.01.


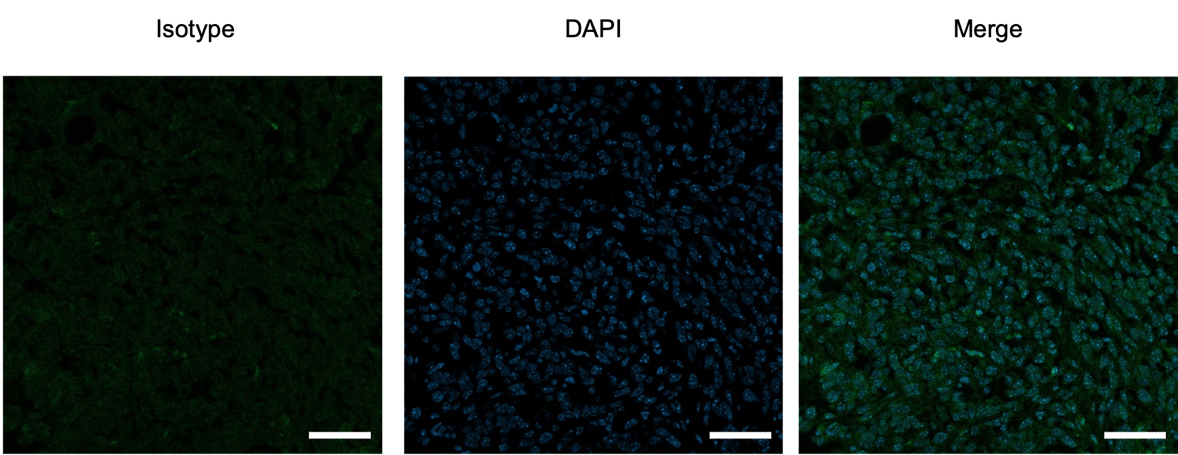


**Fig. S9.** Assessment of nonspecific fluorescence signals in tumor tissues. Rabbit (DA1E) mAb IgG XP® Isotype Control antibody was used to confirm minimal background fluorescence during fluorescent immunohistochemistry. Scale bars represent 50 µm.

**
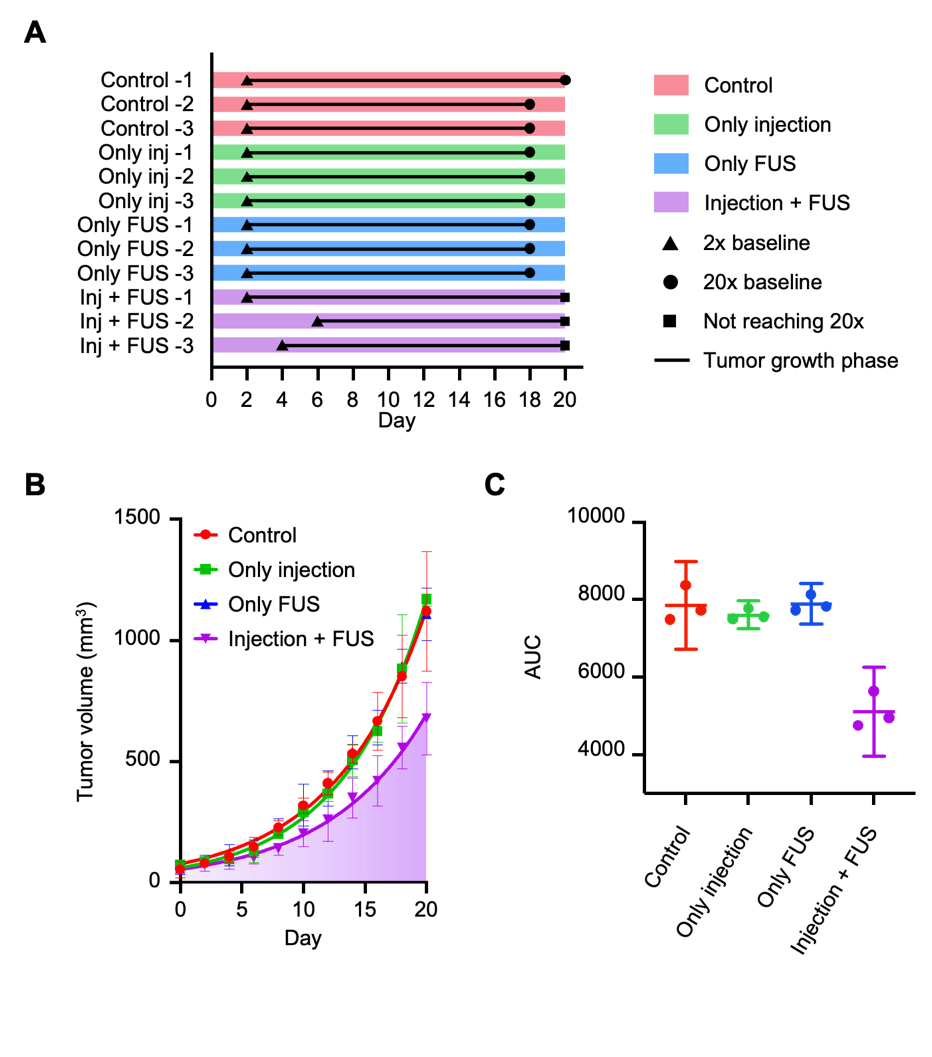
**

**Fig. S10.** Individual-level tumor progression and summary efficacy analyses. (A) Swimmer plot showing time-to-humane-endpoint for individual mice in each treatment group (Control, Only injection, Only FUS, and Injection + FUS). Each horizontal bar represents one animal, with symbols indicating tumor volume thresholds relative to baseline. (B) Tumor growth curves summarized as mean ± 95% confidence interval (CI) over time for each treatment group (n = 3), reflecting uncertainty around the estimated mean trajectories. (C) Area under the tumor volume–time curve (AUC) calculated for each individual animal over the observation period. Points represent individual mice, and bars indicate mean ± 95% CI (n = 3), summarizing cumulative tumor burden using a conservative scalar metric appropriate for small cohort analysis.


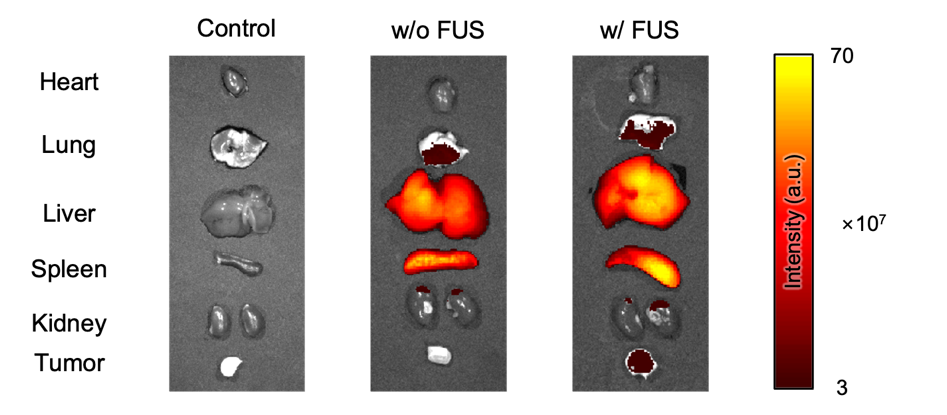


**Fig. S11.** Ex vivo fluorescence imaging of tumors and major organs following intravenous bacterial administration. Representative ex vivo fluorescence images of dissected heart, lung, liver, spleen, kidney, and tumor from mice in the control, without focused ultrasound (w/o FUS), and with focused ultrasound (w/ FUS) groups. Pronounced tumor-associated fluorescence was observed only in the w/ FUS group, whereas minimal tumor signal was detected in the control and w/o FUS groups. Fluorescence signals were consistently observed in the liver and spleen in both w/o FUS and w/ FUS groups, consistent with physiological processing of intravenously administered bacterial carriers by the reticuloendothelial system. In contrast, minimal fluorescence was detected in the heart, lung, and kidney across all groups. Quantitative fluorescence intensity values corresponding to these images are provided in Table S3.

**Supplementary Tables**

**Table S1.** Oligonucleotides used in this work.

| **Name** | **Sequence (5′-3′)** |
| --- | --- |
| ARG_T5_R | GGGTTTCCTGTGTGAAATATTATAATTGTTATCCGCTCACAAAGCAAATAAATTTTTTATGAATTTCGCGGGATCGAGATCTCGATCCT |
| ARG_T5_F | AGCGGATAACAATTATAATATTTCACACAGGAAACCCAGTCGAGCTCATGACTCATATTTATAGTTATTCTCC |
| HCE-trail_R | GAGCTGCTACTCTCTGAGGACCTCTTTCTCTCACCCCGGGATGATGATGATGATGATGCCCCATATGGATATCTCCTTTTTCCAGAAGTG |
| HCE-trail_F | CGGTAAACCAGCAATAGACATAAG |
| Trail_his_F | ATGGGGCATCATCATCATCATCATCCCG |
| Trail_his_R | GCTTATGTCTATTGCTGGTTTACCGGTT |
| Trail_g_R | ACAGCTCCTCGCCCTTGCTAGCGCTCCCACCGCCACTCCCACCGCCGGACCCACCGCCCGACCCACCGCCGCCAACTAAAAAGGCCCCGAAAAAAC |
| Trail_g_F | CAGCAATAGACATAAGCGGCTATTTAACGAC |
| GFP_F | GTCCGGCGGTGGGAGTGGCGGTGGGAGCGCTAGCAAGGGCGAGGAGCTGTTCACCGGGGTGGTGCCC |
| GFP_R | GTCGTTAAATAGCCGCTTATGTCTATTGCTG |

**Table S2.** Strains and plasmids used in this work.

| **Name** | **Relevant characteristics** | **Source** |
| --- | --- | --- |
| **Strains** | | |
| Mach1-T1^R^ | F^-^φ80(*lacZ*)ΔM15 Δ*lacX*74 *hsdR*(r_K_^-^m_K_^+^)Δ*recA*1398 *endA*1 *tonA* | Invitrogen |
| NC | Nissle 1917 | Invitrogen |
| EcN-GVs | EcN/ pET_ARG | This study |
| EcN-TRAIL | EcN/ pCDF-pHCE-TRAIL | This study |
| EcN-GVs+TRAIL | EcN/ pET_ARG/ pCDF-pHCE-TRAIL | This study |
| EcN-GVs+TRAIL-*gfp* | EcN/ pET_ARG/ pCDF-pHCE-TRAIL-*gfp* | This study |
| EcN-*gfp* | EcN/ pACYC-*gfp* | This study |
|  |  |  |
| **Plasmids** | | |
| pET28a_T7_ARG | pET28a_promoterT7_ARG2 | Bourdeau (2018) [37] |
| TRAIL-wt | pET_promoterT7_TRAIL | Rahimizadeh (2022) [25] |
| pCDFDuet | Expression vector, Sm^R^, p15A ori | Novagen |
| pET_ARG | pET28a_promoterT5_ARG2 | This study |
| pACYC-*gfp* | pACYC_promoter J23100_*gfp* | This study |
| pCDF_pHCE-TRAIL | pCDF-promoterHCE-TRAIL- | This study |
| pCDF-pHCE-TRAIL-*gfp* | pCDF_promoterHCE-TRAIL-*gfp* | This study |

**Table S3.** Representative ex vivo fluorescence signal values in tumor and major organs. Values correspond to fluorescence intensity measurements shown in Fig. S11.

|  | **Control** | **w/o FUS** | **w/ FUS** | **Fold change**  **(w/ FUS vs. w/o FUS)** |
| --- | --- | --- | --- | --- |
| Heart | 8.4×10^7^ | 9.5×10^7^ | 1.0×10^8^ | 1.1× |
| Lung | 1.0×10^8^ | 3.7×10^8^ | 4.5×10^8^ | 1.2× |
| Liver | 2.2×10^8^ | 1.2×10^10^ | 1.4×10^10^ | 1.2× |
| Spleen | 9.8×10^7^ | 3.7×10^9^ | 4.6×10^9^ | 1.2× |
| Kidney | 1.5×10^8^ | 2.8×10^8^ | 3.1×10^8^ | 1.1× |
| Tumor | 5.7×10^7^ | 9.3×10^7^ | 1.7×10^8^ | 1.8× |
